# Skin Barrier Biomarkers in Patch‐Induced and Clinical Allergic and Irritant Contact Dermatitis

**DOI:** 10.1111/cod.70070

**Published:** 2025-12-22

**Authors:** Sanja Kezic, Florentine de Boer, Nariman K. A. Metwally, Karen Ghauharali‐van der Vlugt, Femke S. Beers‐Stet, Wouter Ouwerkerk, Ivone Jakasa, Thomas Rustemeyer, Henk F. van der Molen

**Affiliations:** ^1^ Public and Occupational Health Department Amsterdam UMC, University of Amsterdam, Amsterdam Public Health Research Amsterdam the Netherlands; ^2^ Department of Pulmonary Medicine Amsterdam UMC, University of Amsterdam Amsterdam the Netherlands; ^3^ Department of Pediatric Pulmonology and Allergy Emma Children's Hospital, Amsterdam UMC Amsterdam the Netherlands; ^4^ Amsterdam UMC Location University of Amsterdam, Department of Laboratory Medicine and Pediatrics Laboratory Genetic Metabolic Diseases, Emma Children's Hospital Amsterdam the Netherlands; ^5^ Amsterdam Gastroenterology Endocrinology Metabolism, Inborn Errors of Metabolism Amsterdam the Netherlands; ^6^ Core Facility Metabolomics, Amsterdam UMC Location University of Amsterdam Amsterdam the Netherlands; ^7^ Department of Dermatology Amsterdam UMC, University of Amsterdam, Amsterdam Infection and Immunity Institute Amsterdam the Netherlands; ^8^ National Heart Centre Singapore Singapore Singapore; ^9^ Laboratory for Analytical Chemistry, Department of Chemistry and Biochemistry Faculty of Food Technology and Biotechnology, University of Zagreb Zagreb Croatia; ^10^ Dermato‐Allergology and Occupational Dermatology, Amsterdam University Medical Centers Noord‐Holland the Netherlands

**Keywords:** cholesterol derivatives, contact dermatitis, interleukins, natural moisturising factors

## Abstract

**Background:**

Skin barrier impairment is central to irritant (ICD) and allergic contact dermatitis (ACD). Stratum corneum (SC) components cholesterol sulphate (CholSulph), glucosylcholesterol (CholGlc) and natural moisturising factor (NMF) are critical for barrier function, but their changes in ICD and ACD remain underexplored.

**Objectives:**

To measure CholSulph, CholGlc, NMF and IL‐1α in patch‐induced ICD and ACD and in hand dermatitis (HD) diagnosed as ICD or ACD.

**Methods:**

SC samples were collected from HD patients undergoing patch testing. Biomarkers were analysed in positive reactions to sodium lauryl sulphate (ICD, *n* = 44), allergens (ACD, *n* = 113; nickel, chromium, methylisothiazolinone [MI]), lesional HD skin (*n* = 45) and control (empty chamber, *n* = 121).

**Results:**

CholGlc was significantly elevated in patch‐induced ICD and ACD. CholSulph was increased in ICD and chromium‐ and MI‐induced ACD. NMF decreased in ICD, while IL‐1α decreased in ICD and chromium ACD. Chromium induced the strongest response, nickel the weakest. In HD, ICD and ACD showed elevated CholGlc, reduced NMF and IL‐1α, with CholSulph increased only in ACD. No biomarker differences were detected between clinical ICD and ACD.

**Conclusions:**

Both induced and clinical ICD and ACD show consistent SC biomarker changes reflecting barrier dysfunction, with no differences between clinical ICD and ACD.

## Graphical abstract

Both patch‐induced and clinical ICD and ACD showed consistent alterations in skin barrier‐associated biomarkers, indicative of skin barrier impairment. Among the tested allergens, chromium elicited more pronounced biomarker responses compared to nickel and methylisothiazolinone.

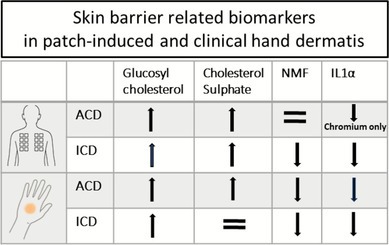

## 1 Introduction

The earliest events following exposure to skin irritants or allergens involve alterations in the composition and organisation of the stratum corneum (SC), the principal barrier of the skin. In irritant contact dermatitis (ICD), cumulative barrier damage triggers innate immune responses, while in allergic contact dermatitis (ACD), barrier disruption facilitates activation of the adaptive immunity [1, 2, 3].

Contact dermatitis (CD) is particularly relevant in occupational settings, where repeated exposure to irritants and allergens frequently leads to occupational contact dermatitis (OCD), most commonly affecting the hands [4, 5, 6]. A major clinical challenge in OCD is distinguishing ICD from ACD, as they often present with the same clinical features. This hampers diagnosis, treatment decisions and the implementation of preventive measures. Recent gene expression and proteomic studies suggest that profiles derived from patch test reactions may help differentiate ICD from ACD and offer potential in subtype prediction in patients with hand dermatitis (HD) [7, 8, 9, 10, 11]. While most investigations have focused on immunological markers from skin biopsies, relatively few studies have examined skin barrier biomarkers in the SC.

Skin barrier integrity depends on the tightly regulated composition of intercellular lipids, primarily ceramides, free fatty acids and cholesterol. These components are modulated by various enzymes, including elongases, ceramide synthases and glucocerebrosidase (GBA), which mediates the conversion of glucosylceramides into ceramides [12]. GBA activity has been shown to increase in atopic dermatitis (AD) [13], a condition associated with higher susceptibility to CD, particularly ICD [14]. Experimental models of skin barrier disruption have also demonstrated upregulation of GBA expression [15]. A notable byproduct of GBA activity is glucosyl cholesterol (CholGlc), which is formed from cholesterol during the enzymatic conversion of glucosylceramides. CholGlc has been found to correlate with transepidermal water loss (TEWL), natural moisturising factors (NMF) and interleukin‐1α (IL‐1α), all established indicators of skin barrier status [13]. NMF, derived from filaggrin breakdown, is essential for SC hydration and pH regulation, which in turn may influence GBA activity [6].

Cholesterol sulphate (CholSulph), another cholesterol derivative, plays a key role in regulating keratinocyte differentiation and desquamation and is produced via cholesterol sulfotransferase [16]. Like CholGlc, CholSulph is elevated in AD and may be involved in barrier‐related inflammatory processes [16, 17]. In contrast to ceramides that have been extensively studied in skin barrier dysfunction, cholesterol derivatives remain underexplored in CD, and their difference between ICD and ACD is unclear.

In the present study, we investigated levels of CholSulph, CholGlc, NMF and IL‐1α in patch‐induced ICD and ACD, as well as in lesional skin of patients with HD. These biomarkers were selected due to their relevance to skin barrier function, a key element in both ICD and ACD, albeit through distinct mechanisms. Our aim was to better understand their role in CD pathophysiology and explore their potential utility in distinguishing between ICD and ACD in patients with occupational HD.

## 2 Methods

### 2.1 Study Participants

A total of 153 patients with HD were enrolled at the Occupational Dermato‐Allergology Clinics of Amsterdam UMC during routine allergen patch testing. Patients receiving systemic therapy or presenting with other inflammatory skin diseases were excluded. All participants were instructed to refrain from topical therapy for at least 72 h prior to patch testing.

From these participants, 323 SC samples were collected for analysis. The study protocol complied with the principles outlined in the Declaration of Helsinki and was approved by the Ethics Committee of Amsterdam UMC (reference number W23_108#23.132). Written informed consent was obtained from all participants.

### 2.2 Patch Testing and Diagnostics of HD

Patch testing was carried out according to the guidelines of the European Society of Contact Dermatitis [18]. Allergen preparations (Smart Practice Europe, Greven, Germany) were applied to the upper back using AllergEAZE clear test chambers (Smart Practice Europe). A control was an empty chamber. Patches were removed on Day 3 (D3), and skin reactions were evaluated on Day 4 (D4) using a standardised scoring system (+, ++, +++). Diagnosis of HD was made by the treating physician and was based on a combination of clinical examination, patch test results and exposure history. Only cases with a unique subtype (either ICD or ACD) were included. Patients with mixed aetiologies (e.g., ICD combined with ACD, or overlap with AD) were excluded. In the study, only patients with weak‐positive (+) and strong positive (++) reactions were included; patients with +++ reactions were excluded, as such intense responses involve confluent vesicles with exudation, which may interfere with assessment and make tape stripping burdensome for the patient.

### 2.3 Stratum Corneum Tape Strip (SCTS) Sampling

**FIGURE 1:**
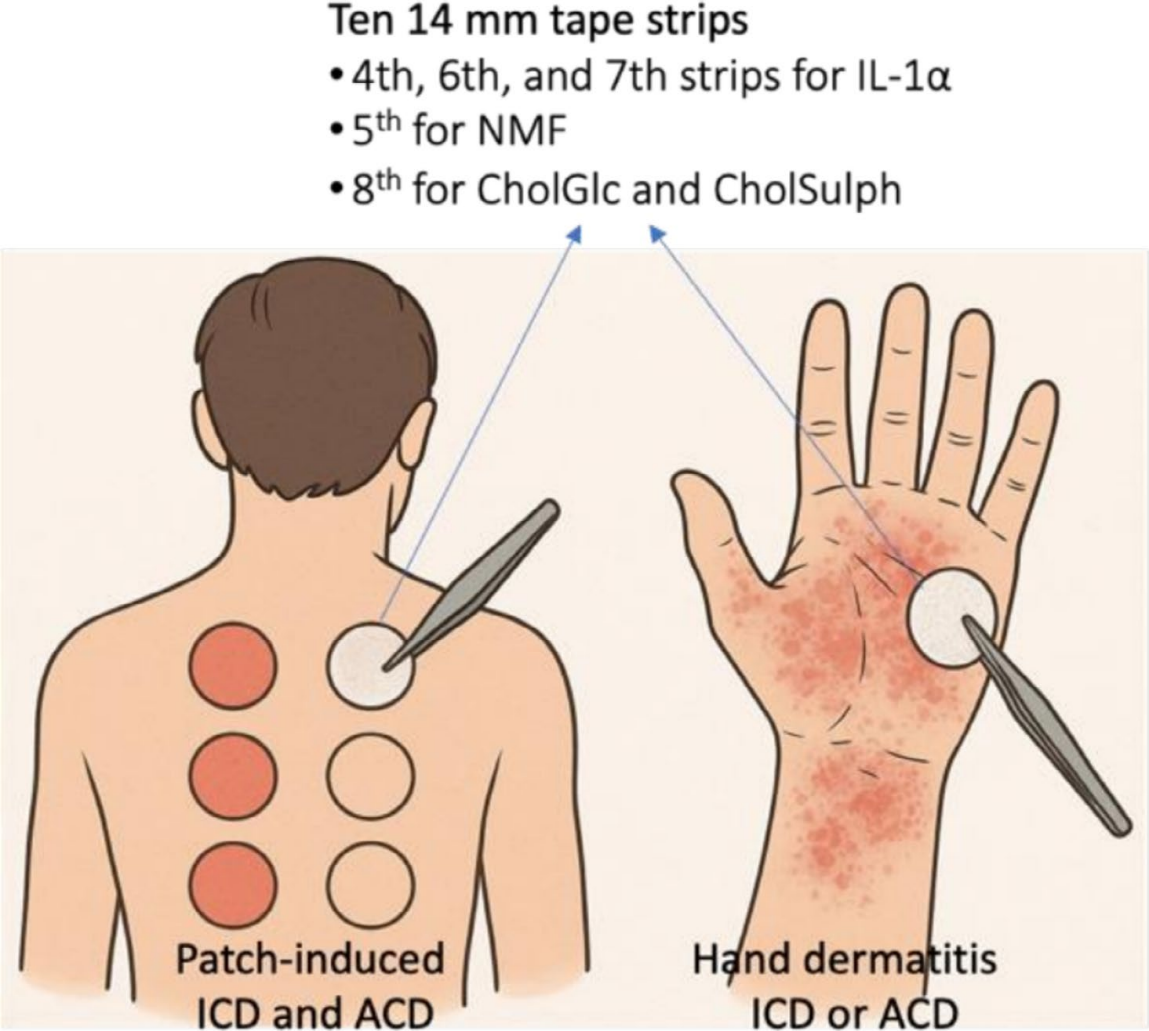
Stratum corneum tape strip (SCTS) sampling. SCTS were collected from positive patch test sites on the upper back skin (ACD: Ni, Cr, MI; ICD: SLS) with empty chambers as controls, and from lesional skin of hand dermatitis. Ten sequential 14‐mm strips (D‐Squame) were obtained per site; selected strips were analysed for IL‐1α, NMF, CholGlc and CholSulph. Created by ChatGPT‐4 with DALL·E (OpenAI, San Francisco, CA, USA), accessed July 2025.

SCTS were collected at the time of patch reading, 96 h after patch application (D4). Two types of SCTS samples were obtained (Figure 1). First, SCTS were obtained from the sites of positive patch test reactions to one of the allergens, including nickel (Ni), chromium (Cr), methylisothiazolinone (MI) and sodium lauryl sulphate (SLS) as a model irritant, along with a control sample (empty chamber). Second, SCTS were collected from the hands of patients presenting with HD. Samples from positive patch reactions to allergens (Ni, Cr or MI) were classified as ACD, while those responding to SLS were classified as ICD. In HD, SCTS were collected from the affected skin areas of the dorsal or palmar hand.

To collect SCTS, round‐shaped adhesive tape strips (14 mm diameter; D‐Squame, CuDerm, Dallas, TX) were placed on the skin site and pressed with uniform pressure for 5 s, following protocols described in detail elsewhere [19]. Each strip was then removed using tweezers and placed into individual vials, which were stored at −80°C until further processing. A total of 10 sequential strips were collected from each skin site. The fourth, sixth and seventh strips were analysed for IL‐1α, the fifth for NMF and the eighth for CholGlc and CholSulph.

### 2.4 Biomarker Analysis

CholGlc and CholSulph were extracted from the tape strips using a methanol–chloroform–water mixture (12:6:1, v/v/v), and the extract was dried under nitrogen at 40°C, as described previously [13]. Following the addition of internal standards glucosylstigmasterol and cholesterolsulphate‐*d*_7_, phase separation was induced with methanol–chloroform–water (1:1:0.9, v/v/v). The lower phase was collected in a 2 mL tube, while the upper phase was washed with chloroform. The combined lower phases were then dried (N_2_, 40°C), and the residues were reconstituted in 150 μL of 10 mmol L^−1^ ammonium formate in methanol for LC–MS/MS analysis. To correct for the variable amounts of SC on the tape, concentrations of CholGlc and CholSulph were normalised by protein concentration measured on each tape using SquameScan optical densitometer 850 A (CuDerm, Dallas, TX).

The analysis of NMF was performed by a previously described high‐performance liquid chromatography method [20]. NMF was normalised by protein amount, determined by the Pierce Micro BCA Protein Assay Kit (Thermo Fisher Scientific, Rockford, IL, USA).

The method for IL‐1α determination is described in detail elsewhere [13]. Briefly, to the fourth tape strip, 0.8 mL of phosphate‐buffered saline (PBS; Merck, Darmstadt, Germany) containing 0.05% Tween 20 (Sigma‐Aldrich, Zwijndrecht, The Netherlands) was added and sonicated for 15 min in an ice bath (Branson 5800, Ede, The Netherlands). The extract was then reused to extract the sixth tape and then again applied to the seventh tape. Cytokine concentrations in the extracts were measured on multiplex panels using MESO QuickPlex SQ 120 (MSD, Rockville, MA, USA). The amount of IL‐1α was normalised by protein amount, determined in the extracts using the Pierce Micro BCA Protein Assay Kit.

### 2.5 Statistical Analysis

Baseline characteristics were summarised by mean (SD), median (25th–75th percentile) or number (percentages). Continuous data were compared using independent *t*‐tests for normally distributed data, or the Mann–Whitney *U* test for non‐normally distributed data. Binary data were compared with Pearson's *χ* ^2^ test or Fisher's exact test, dependent on the sample size.

We log‐transformed all biomarker values.

We estimated the Pearson's correlation coefficients between IL‐1α, NMF and cholesterols.

Since not all patients have equal skin condition measurements or patch tests, we developed a mixed effects model, with patients as random effects, estimating biomarker levels within the different skin conditions (patch test control, lesional HD skin, ICD and ACD), correcting for age and sex. We also developed a patch‐specific mixed effects model where the different patch tests (Chromium, MI and Nickel) were evaluated, along with the control and ICD patch, correcting for age, sex and patch test reaction. In presenting the differences between ACD and ICD versus control and the protein levels from the different panels, we used the model estimates to calculate protein distributions. The fixed effects distribution is therefore based on model estimates and does not represent the actual individual patient protein data. We have done this by modelling a normal distribution around the mean and SE. The interquartile range (IQR) is based on the 75th percentile–25th percentile calculated by the qnorm() function in R (R Core Team version 4.4.1). This IQR is used to estimate the upper and lower whiskers by 25th/75th percentile ±1.5 × IQR.

A two‐sided, Benjamini–Hochberg multiple‐testing corrected *p* < 0.05 was considered statistically significant. All analyses were performed in R version 4.4.1.

## 3 Results

A total of 138 patients were included in the study; 75.4% were female. SCTS samples were collected from 22 patch test sites on the back skin, positive for chromium (Cr), 51 for nickel (Ni), 40 for MI and 44 for SLS. Among patients with a positive allergen patch test, the distribution of reaction intensities differed between allergens. For chromium, 95% showed a weak positive reaction (+) and 5% a strong positive reaction (++). For MI, 85% were weak positive (+) and 15% strong positive (++). For nickel, 82% were weak positive (+) and 18% strong positive (++).

If a patient had positive reactions to multiple allergens or to both an allergen and SLS, SCTS samples were collected from each individual reaction site. Consequently, multiple samples were obtained from some patients, corresponding to different types of skin responses.

Samples from HD lesions were obtained from 45 patients, of whom 64.4% were diagnosed with ACD and 35.6% with ICD. The majority of HD lesions were located on the dorsal side of the hands (55.6%).

Demographic and clinical characteristics of the study population are summarised in Table 1.

**TABLE 1:** Demographic characteristics of study population.

Characteristic	*n* (%)
Sex	
Female	104 (75.4%)
Male	34 (24.6%)
Age, years	42.6 (16.9)
Patch test reactions allergens (*n* = 113)	
Chromium: Potassium dichromate, 0.5% in petrolatum (Allergeaze, Reinbek, Germany)	22 (19.4%)
Methylisothiazolinone, 0.2% in water (Allergeaze, Reinbek, Germany)	40 (35.4%)
Nickel sulphate, 5% in petrolatum (Allergeaze, Reinbek, Germany)	51 (45.1%)
Patch test reactions Sodium lauryl sulphate, 2% in water (Dermatology Laboratory Amsterdam UMC)	44
Patch control (empty chamber)	121
Hand dermatitis	45
ACD	29 (64.4%)
ICD	16 (35.6%)
Hand dermatitis location	
Dorsal side	25 (55.6%)
Palmar side	20 (44.4%)

### 3.1 Differences in Biomarker Levels Between Patch‐Induced ICD, ACD and Control Skin

**FIGURE 2:**
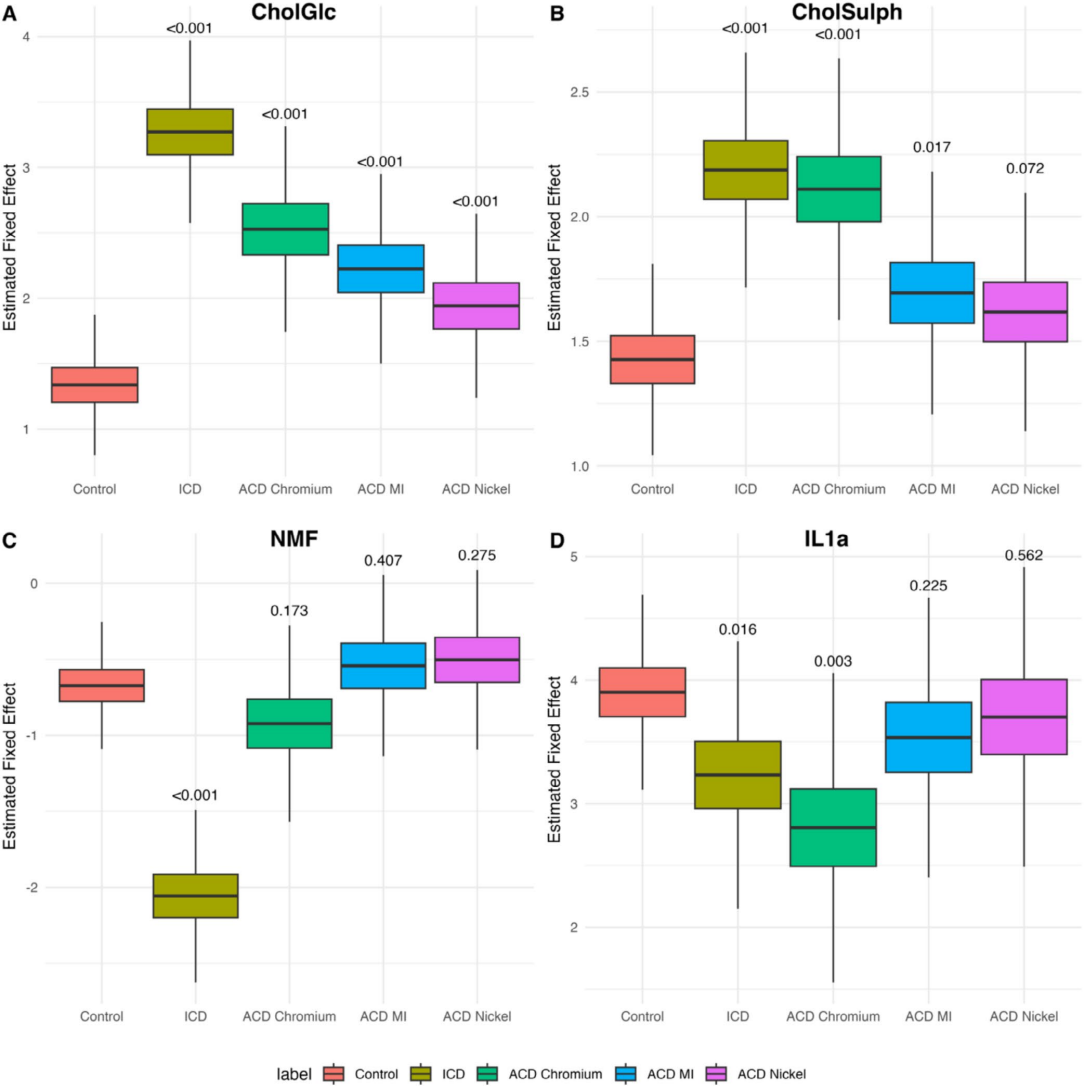
Estimated fixed effects for stratum corneum biomarkers comparing control skin (empty chamber) with patch‐induced irritant contact dermatitis (ICD) and allergic contact dermatitis (ACD). All samples were obtained from the upper back at patch test sites. (A) Glucosyl cholesterol (CholGlc); (B): cholesterol sulphate (CholSulph); (C): natural moisturising factors (NMF) and (D) interleukin‐1α (IL‐1α). Differences from control (*n* = 121) were assessed using linear mixed‐effects models with patient ID as a random effect and fixed effects for condition (control, ICD, ACD), adjusted for age, sex and patch test reaction. ICD was induced by sodium lauryl sulphate (SLS, *n* = 44) and ACD by chromium (*n* = 22), methylisothiazolinone (MI, *n* = 40) and nickel (*n* = 51). *p*‐Values for comparisons with control are shown above the boxes. Boxplots illustrate modelled fixed effect distributions, not raw patient data.

Mixed effects models identified consistent biomarker changes between control skin, ICD and ACD, all sampled from the upper back. Table S1 presents the numerical model estimates (effect size ± SE, and *p*‐value), while Figure 2 visualises the corresponding modelled fixed effect distributions, which do not represent raw patient data.

CholGlc was significantly increased in all dermatitis groups. According to the model estimates (Table S1), ICD showed the strongest effect (1.93 ± 0.17, *p* < 0.001), followed by ACD Chromium (1.19 ± 0.21, *p* < 0.001), ACD MI (0.89 ± 0.18, *p* < 0.001) and ACD Nickel (0.60 ± 0.17, *p* < 0.001). The modelled distributions confirmed elevated CholGlc across all dermatitis groups compared with controls (Figure 2A).

CholSulph was significantly elevated in ICD (0.76 ± 0.10, *p* < 0.001) and ACD Chromium (0.68 ± 0.13, *p* < 0.001), with weaker effects observed in ACD MI (0.27 ± 0.11, *p* = 0.017) and a non‐significant trend in ACD Nickel (0.19 ± 0.11, *p* = 0.072) (Table S1). The modelled distributions reflected the same pattern, with the clearest increases in ICD and Chromium ACD (Figure 2B).

NMF was markedly reduced in ICD (−1.38 ± 0.14, *p* < 0.001), while no significant changes were observed for ACD Chromium, MI or Nickel (all *p* > 0.17) (Table S1). The modelled distributions showed that this reduction was specific to ICD (Figure 2C).

IL‐1α was decreased in ICD (−0.67 ± 0.27, *p* = 0.016) and ACD chromium (−1.10 ± 0.36, *p* = 0.003), but not significantly altered in ACD MI (−0.37 ± 0.30, *p* = 0.225) or Nickel (−0.20 ± 0.34, *p* = 0.562) (Table S1). The modelled distributions supported these reductions for ICD and chromium ACD (Figure 2D).

Overall, ICD was characterised by pronounced increases in CholGlc and CholSulph, together with reductions in NMF and IL‐1α. ACD responses were more variable, with chromium eliciting the strongest allergen‐associated changes, while MI and nickel produced moderate effects limited mainly to CholGlc (and for MI, CholSulph).

### 3.2 Differences in Biomarker Levels Between Different Types of HD and Control Skin

**FIGURE 3:**
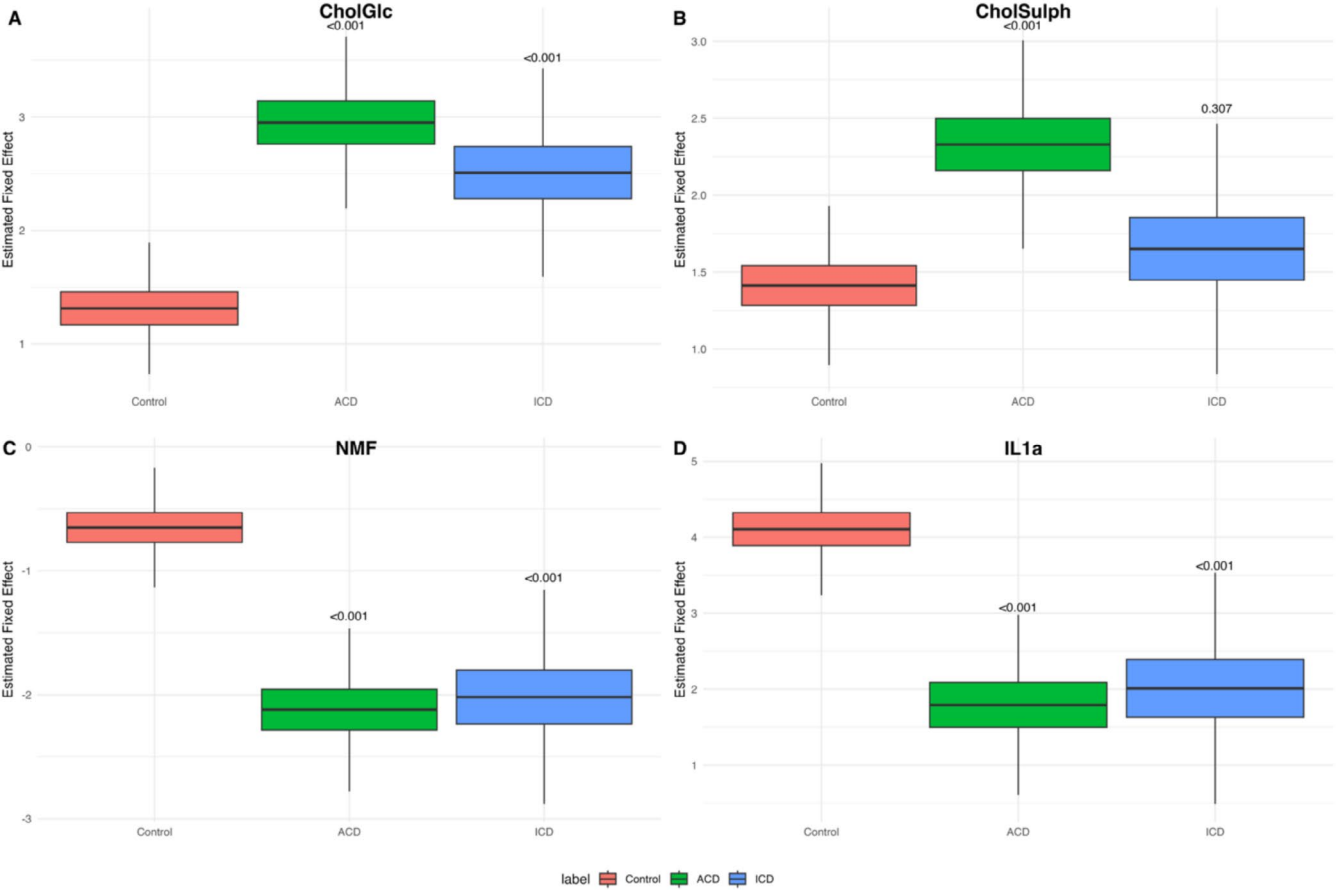
Estimated fixed effects for stratum corneum biomarkers comparing control skin with hand dermatitis diagnosed as irritant contact dermatitis (ICD) or allergic contact dermatitis (ACD). (A) Glucosyl cholesterol (CholGlc); (B) cholesterol sulphate (CholSulph); (C) natural moisturising factors (NMF) and (D) interleukin‐1α (IL‐1α). Control samples were obtained from the upper back at patch test sites (empty chamber).

We next analysed biomarker changes in patients with clinically diagnosed HD, comparing ICD and ACD with control skin (empty chamber on back skin) (Table S1 and Figure 3). Model outputs are reported as estimate ±SE and *p*‐value.

Mixed effects models demonstrated distinct biomarker alterations in HD compared with control skin.

CholGlc was significantly elevated in both ACD (2.95 ± 0.28, *p* < 0.001) and ICD (2.51 ± 0.34, *p* < 0.001) compared with controls (1.31 ± 0.21). The modelled fixed‐effect distributions confirmed these increases, with ACD showing the highest levels (Figure 3A).

CholSulph was significantly increased in ACD (2.33 ± 0.25, *p* < 0.001), whereas ICD (1.65 ± 0.30, *p* = 0.307) showed no significant difference from control (1.41 ± 0.19) (Table S1). The modelled distributions reflected this pattern (Figure 3B).

NMF was markedly reduced in both ACD (−2.12 ± 0.24, *p* < 0.001) and ICD (−2.02 ± 0.32, *p* < 0.001) compared with controls (−0.65 ± 0.18). The modelled distributions showed a consistent pattern across both conditions (Figure 3C).

IL‐1α was decreased in ACD (1.79 ± 0.44, *p* < 0.001) and ICD (2.01 ± 0.56, *p* < 0.001) relative to controls (4.11 ± 0.32). The modelled distributions supported these reductions, with the lowest values observed in ACD (Figure 3D).

No significant differences were observed between ICD and ACD for any of the investigated biomarkers (*p* > 0.05).

Differences from control were assessed using linear mixed‐effects models with patient ID as a random effect and fixed effects for condition (control, ICD, ACD), adjusted for age, sex and patch test reaction. *p*‐Values for comparisons with control are shown above the boxes. Boxplots illustrate modelled fixed‐effect distributions, not raw patient data.

### 3.3 Correlations Between Biomarkers

**FIGURE 4:**
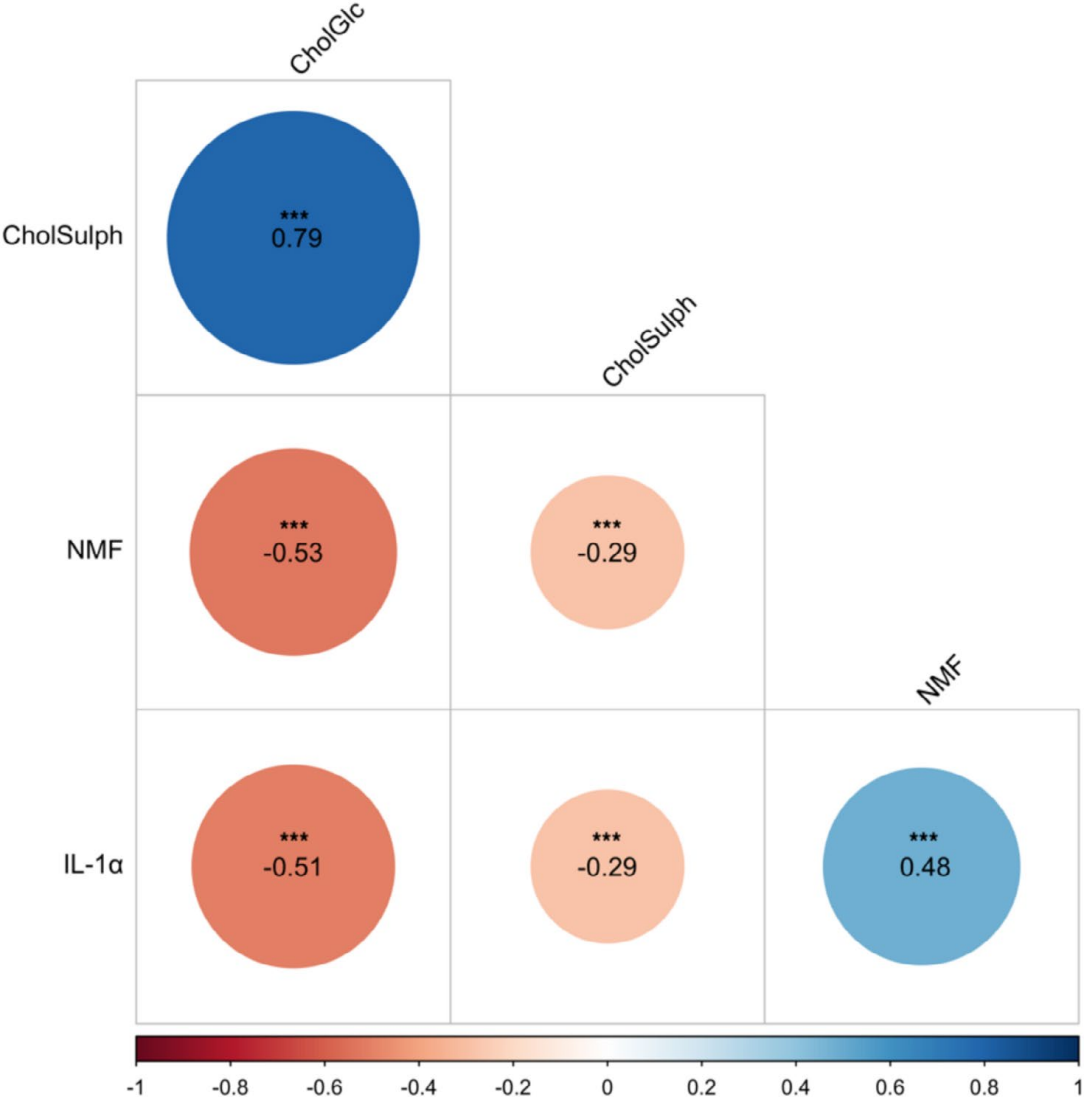
Pearson correlation matrix of glucosyl cholesterol (CholGlc), cholesterol sulphate (CholSulph), natural moisturising factors (NMF) and IL‐1α. Circle size and colour reflect the strength and direction of the correlation coefficient (*r*). Asterisks above correlation coefficients indicate significance; here, ***p < 0.001 for all correlations.

Pairwise Pearson correlations between the four biomarkers are shown in Figure 4. A strong positive correlation was found between CholGlc and CholSulph (*r* = 0.79, *p* < 0.001). Moderate negative correlations were observed between CholGlc and NMF (*r* = −0.53, *p* < 0.001) and IL‐1α (*r* = −0.51, *p* < 0.001). Significant, moderate positive correlation was found between NMF and IL‐1α (0.48, *p* < 0.001).

## 4 Discussion

This study demonstrates that CD, whether experimentally induced or clinically manifested, is consistently associated with alterations in SC biomarkers that reflect skin barrier dysfunction. These findings not only confirm the involvement of barrier‐related molecules in acute experimental CD but also extend their relevance to clinical HD.

### 4.1 Experimentally Induced ICD and ACD

Although cholesterol derivatives are essential for the epidermal barrier, they have been rarely studied in CD. CholSulph, the most abundant cholesterol derivative in the SC, promotes keratinocyte differentiation and influences SC desquamation and lipid lamellae organisation, thereby affecting barrier function [21, 22, 23]. We found increased CholSulph levels in ICD and in chromium‐ and MI‐induced ACD, with only a non‐significant tendency in nickel ACD. Chromium induced the largest response among allergens, followed by MI and nickel. Elevated CholSulph levels have also been reported in AD [17], a common inflammatory skin disorder associated with impaired skin barrier and a known risk factor for ICD [14, 24].

Notably, recent research has identified CholSulph as a ligand for the C‐type lectin receptor Mincle (Clec4e), which is upregulated in response to skin injury and irritation [25, 26]. Upon binding CholSulph, Mincle activates downstream proinflammatory signalling pathways and promotes the recruitment of antigen‐presenting cells. This suggests that elevated CholSulph levels may contribute to the pathogenesis of ACD by both impairing barrier function and amplifying cutaneous immune responses through Mincle signalling.

CholGlc, formed from cholesterol by GBA [27], was also elevated after both ICD and ACD. Although the precise function of CholGlc remains unclear, its hydrophilic properties may affect lipid organisation and consequently skin barrier function. We previously demonstrated increased GBA activity in AD and a strong correlation between CholGlc levels and skin barrier function assessed as TEWL [13]. Notably, recent studies indicate that CholGlc, like CholSulph, may be recognised by Mincle, suggesting its role in skin immune responses [28].

In line with previous findings in AD, CholGlc levels were inversely correlated with IL‐1α, which was significantly reduced in ICD and chromium‐induced ACD. IL‐1α, a key proinflammatory cytokine constitutively produced by keratinocytes and stored in the SC, is rapidly released upon barrier damage to initiate skin barrier repair [29, 30]. The observed decrease in IL‐1α may reflect the depletion of the preformed pool due to barrier damage. Reduced IL‐1α levels have been reported in response to various irritants and allergens, underscoring its role in early immune responses in both ICD and ACD [19, 20, 31].

NMF, a complex mixture of water‐soluble compounds primarily derived from the degradation of epidermal protein filaggrin, was significantly reduced only in ICD. Decreased NMF levels in ICD induced by various skin irritants have previously been demonstrated in various studies [19, 20, 31, 32]. Although the exact mechanisms underlying its reduction remain unclear, possible pathways include decreased filaggrin expression, altered proteolytic processing from filaggrin or leakage from corneocytes due to changes in structural proteins of corneocyte envelopes.

Among the tested allergens, chromium consistently induced the most pronounced biomarker changes, while nickel elicited the weakest responses. This likely reflects differences in irritant potency of the allergens, as chromium exerts strong irritant effects in addition to its allergenic potential. In an in vitro study, chromium was significantly more cytotoxic than nickel and induced higher levels of IL‐1α [33]. The overlap of irritant and allergic reactions complicates the interpretation of biomarker changes and underscores the shared pathways of barrier disruption and inflammation. Notably, however, the strongest biomarker changes observed for chromium do not mirror the patch test outcomes, as chromium showed the lowest proportion of strong positive reactions (5% ++), compared with 15% for MI and 18% for nickel.

### 4.2 HD

Our findings demonstrate that biomarker alterations in clinical ICD and ACD closely parallel those seen in the patch test model. Both ICD and ACD showed elevated CholGlc and reduced NMF and IL‐1α, while CholSulph was increased only in ACD. Notably, while ICD responses were consistently more pronounced than ACD in patch testing, in clinical CD there was no significant difference between ICD and ACD. Several factors may explain the smaller differences between ICD and ACD in HD compared with patch testing. In patch testing, ICD is induced by acute exposure to a strong irritant (SLS), producing rapid and severe barrier disruption [34]. By contrast, chronic ICD often develops from repeated low‐level exposures (e.g., occupational wet work), where compensatory mechanisms may partially restore barrier integrity and dampen biomarker responses. ACD, meanwhile, is elicited by allergens that cause less acute disruption in patch testing, but progressive barrier deterioration may accumulate in chronic disease. In chronic ACD, Th2‐skewed inflammation may also arise, and Th2 cytokines are known to downregulate filaggrin expression, further weakening the skin barrier [35, 36, 37].

Exposure‐related factors may also contribute. The culprit allergen in HD is expected to affect the biomarker response in ACD; for example, if chromium accounted for many cases, overall responses may have been amplified, as chromium produced the strongest changes in patch testing. In addition, real‐life exposures are often stronger or more prolonged than standardised patch testing, which can further amplify irritant‐driven changes in ACD and blur its distinction from ICD.

### 4.3 Limitations

This study has several limitations. First, the specific causative allergens in patients with clinical HD were not identified, precluding allergen‐specific comparisons. Second, tape strip samples were collected from different hand sites. Although Sølberg et al. [11] demonstrated that SC transcriptomic profiles are largely consistent across hand locations, site‐related variability cannot be fully excluded. Third, disease severity in HD was not assessed, which may have influenced biomarker expression and contributed to heterogeneity within the ICD and ACD groups. Finally, potential misclassification in HD and the lack of non‐lesional samples from the hands as controls limited the possibility of matched paired analyses in HD.

## 5 Conclusion

This study shows that both patch‐induced and clinical CD are characterised by consistent SC biomarker alterations reflecting barrier dysfunction. Irritants and allergens produce largely overlapping patterns, with chromium eliciting the strongest responses among allergens. These findings emphasise shared pathways of barrier impairment in ICD and ACD and underscore the need for further studies on how different exposures contribute to barrier damage and chronic disease.

## Author Contributions

**Sanja Kezic:** conceptualization, investigation, supervision, data curation, writing – review and editing. **Florentine de Boer:** conceptualization, investigation, supervision, data curation, writing – review and editing. **Nariman K. A. Metwally:** investigation, writing – review and editing, data curation. **Karen Ghauharali‐van der Vlugt:** biomarker analysis, review and editing. **Femke S. Beers‐Stet:** biomarker analysis, review and editing. **Wouter Ouwerkerk:** formal analysis, data curation, writing – review and editing, methodology. **Ivone Jakasa:** biomarker analysis, review and editing. **Thomas Rustemeyer:** conceptualization, investigation, writing – review and editing, supervision. **Henk F. van der Molen:** conceptualization, investigation, writing – review and editing, supervision.

## Funding

This work was supported by Lexces. The expertise centre for the prevention and assessment of occupational diseases caused by exposure to hazardous substances.

## Disclosure

A preliminary analysis of a subset of the cohort, addressing related but more limited research questions, has been published previously (Grob et al. Clinics and Practice, 2025, 15(12), 217). The current study analyzes the full cohort and extends the biomarker panel and statistical analyses.

## Conflicts of Interest

The authors declare no conflicts of interest.

## Supporting information


**Table S1:** Model estimates for hand dermatitis and patch test data.

## Data Availability

The data that support the findings of this study are available from the corresponding author upon reasonable request.
